# The ecological fitness of the tomato potato psyllid after transferring from non-crop host plants to tomato and potato

**DOI:** 10.1371/journal.pone.0266274

**Published:** 2022-04-07

**Authors:** Howard London, David J. Saville, Melanie M. Davidson, Oluwashola Olaniyan, Stephen D. Wratten

**Affiliations:** 1 Department of Ecology, Bio-Protection Research Centre, Lincoln University, Lincoln, Canterbury, New Zealand; 2 Plant, Pathology, Entomology and Weed Science Department, National Agricultural Research and Extension Institute, Mon Repos, East Coast Demerara, Guyana; 3 Saville Statistical Consulting Limited, Lincoln, Canterbury, New Zealand; 4 Beneficial Biodiversity team, Plant & Food Research, Lincoln, Canterbury, New Zealand; University of Saskatchewan College of Agriculture and Bioresources, CANADA

## Abstract

An insect’s fitness varies on different host plant species, and can be affected by previous host feeding experience. In New Zealand, *Bactericera cockerelli* (the tomato potato psyllid (TPP)) overwinter on various host species, and later migrate to annually grown crop host plants. How changing host plant species affects the insect’s fitness is unknown. This study evaluated if transferring adult TPP from non-crop to crop host species impacts the development and survival of their progeny. TPP were reared on non-crop host species, boxthorn, poroporo, and crop host species, potato and tomato. Adults were transferred from non-crop to the crop host species and allowed to oviposit for 48 hours before being removed. The eggs and nymphs were monitored every 24 hours for the development and survival of each life stage. The incubation period of eggs from adults transferred from poroporo to tomato was 6.9 days, and for boxthorn to tomato was 7.2 days, and was less than for eggs of adults moved from tomato to tomato (9.0 days) and potato to potato (9.2 days) (P < 0.05). Nymph developmental time was similar for all treatments. Total development time (egg to adult) was shorter for the progeny of adults from poroporo transferred to tomato (20.5 days) than those from tomato to tomato (23.2). The survival of eggs did not differ across treatments. Fewer nymphs survived when adults were transferred from tomato to tomato (50.4%) than those from poroporo to tomato (92.1%) (P < 0.05). Total survival (egg to adult) was higher for progeny of adults transferred from poroporo to tomato (80.0%) compared to boxthorn to potato (35.3%), boxthorn to boxthorn (40.7%), poroporo to potato (33.9%) and tomato to tomato (37.6%) (P < 0.05). The implications of this shift in fitness are discussed in relation to TPP management.

## Introduction

The intrinsic rate of increase of a polyphagous herbivorous insect is influenced by its host plant [1]. Host plant selection is determined by ecological [2, 3] and nutritional factors [4, 5] and fertility, survival and development are likely to vary accordingly. Because immature life stages of *Bactericera cockerelli*, the tomato potato psyllid (TPP) are mostly immobile [6], the host plant chosen by their mother has to be suitable for their development and survival to maturity [1]. However, not all host plant species are equally suitable. Survival of TPP eggs and nymphs differed significantly when reared on *Solanum tuberosum* L., *Ipomoea batatas* (L.) Lam. and *Solanum aviculare* (G. Forst) in a glasshouse experiment [7], and the development rate on *S*. *tuberosum* and *Solanum elaeagnifolium* Cav. were reported to be significantly different [8]. Therefore, the ecological fitness was affected by the host plant species.

Some parameters that can be measured as proxies for fitness include; growth rate and survival of immature life stages (i.e., eggs and nymphs), and sex ratio, body size and fecundity of adults [9–11]. Therefore, the concept adopted for this research is the measurement of how well TPP adapts to a host plant [10, 11]. Some known host plants of TPP found in the Canterbury region of New Zealand are; African boxthorn (*Lycium ferocissimum* Miers) introduced as a hedgerow species to coastal and lowland areas [1, 12–14], the widely distributed native shrub, poroporo (*Solanum aviculare* G. Forst.) [1, 13–15], the widespread accidentally introduced herbaceous weed species field bindweed (*Convolvulus arvensis* L.) [1, 13, 14] and black nightshade (*Solanum nigrum* L.) [16, 17], and the commonly grown crop species potato (*Solanum tuberosum* L.) [7, 15, 18, 19] and tomato (*Solanum lycopersicum L*.) [15, 20, 21], with commercial tomato crops grown in glasshouses in Canterbury.

Previous studies have shown that TPP’s ecological fitness differs on different host plant species. However, changes in the ecological fitness when TPP adults move from perennial non-crop hosts (e.g. African boxthorn or poroporo) to annual crop host species in spring and summer is unknown. Some host plants are only available seasonally, while others are suitable hosts all year round and serve as overwintering hosts [6],It is essential to understand if the insect progeny suffer changes in ecological fitness after adults transfer from one host plant species to another, given the role different host plant species play at different times of the year [6, 22]. This information can explain the pest’s possible intrinsic rate of increase when they move between host species. Knowing the intrinsic rate of increase is imperative in planning possible management strategies for the pest. This study evaluated if transferring adult TPP from non-crop to crop host species impacts the development and survival of their progeny.

## Materials and methods

### Plants

Potato (Ilam Hardy G6, a common cultivar in New Zealand) was grown in a glasshouse at the Plant Nursery, Lincoln University, and tomato plants (Merlice, a common cultivar in New Zealand) were sourced from Zealandia Horticulture (https://www.zealandia.co.nz), a commercial seedling producer. The plants were planted in 0.75 litre planter pots filled with a potting mix comprised of 400L compressed bark, 1500g of Osmocote 3–4 months release (http://www.farmcraft.com.au/), 500g horticultural lime, 500g of hydraflo and 100L pumice. Cuttings from perennial boxthorn and poroporo, and the annual species field bindweed and black nightshade were collected from plants in areas around Lincoln University in Canterbury, New Zealand. The age of these plants were unknown, however, the boxthorn and poroporo were likely to be several years old given the size of the plants. These plant species were selected because they are hosts of TPP [1, 13–15, 18, 20, 21]. Cuttings were treated with Rooting hormone (Murphy’s), placed in planting trays with Perlite and situated in a warm, humid glasshouse to root. Periodically the cuttings were supplied with water mist to keep them from drying out. After rooting, the cuttings were potted similarly to the tomatoes and potatoes. All plants were situated in the glasshouse for further development and supplied with water as required. The non-crop host plants were kept for approximately 90 days, while the crop host plants were approximately 35 days old.

### Psyllid culture

TPP adults were collected from boxthorn in the wild and from a colony kept on potato plants (cultivar Swift) at the plant nursery, Lincoln University. Colonies were then established by placing 20 adult TPP each on tomato, potato, poroporo, field bindweed, black nightshade, and boxthorn plants kept in separate "BugDorm" -2 Rearing Cages (L60 x W60 x H60 cm and Mesh Size of 680 μm opening, MegaView Science Company, Limited, Taiwan). The colonies were kept in a controlled temperature (CT) room at 23°C with a 4°C range at 60% RH, and a photoperiod of 16:8 (L:D) h, because these are the best conditions for the insect’s growth and development [23]. New healthy plants were added to the colonies as older plants began to senesce, and older plants were eventually removed after complete senescence. Plants were watered as needed.

The insect culture did not establish on field bindweed and black nightshade, despite several attempts at infesting these two plants species with TPP. It was evident that the insect was feeding and reproducing on the plants because, eggs, early instar nymphs and psyllid sugars were observed, but later instar nymphs and newly emerged adults were never observed. The insect was unable to survive through the immature life stages or complete an entire life cycle on these plants. Thus, further experiments with these plant species were not undertaken.

At Plant and Food Research, Lincoln, a subsample of TPP collected from the different host plants were screened for the presence of the bacteria *Candidatus* Liberibacter solanacearum [20] (CLso). PrepGEM^TM^ reagents were used to extract DNA from the insect in preparation for CLso measurement using qPCR [24].

The survival of TPP bearing CLso can be affected by the presence of the pathogen [25]. Psyllid samples from all TPP colonies used in this study tested positive for CLso. This indicates that the presence of CLso had no biasing effect on the survival of the insect. Haplotype may also influence an insect’s response to a host plant [26]. However, in New Zealand, only one haplotype (western) has been detected [27].

### Experimental setup

Adults were collected from colonies with the aid of an aspirator and separated into males and females by observing the apex of the abdomen under a binocular microscope. The male apex is pointed while that of the female is rounded and robust and has a short ovipositor [28]. Four pairs of nine day old adults were placed on plants (Table 1) covered by Vienna hot micro-perforated bread bags with dimensions of 240(W) x 420(H) +75(G)mm and thickness of 25μm. Adults were removed after 48 h, and the number of eggs per plant was recorded. Plants containing no more than 36 eggs were used for observation. There were six replicates per treatment; each plant was a replicate. Plants were placed in a random order in six randomised complete blocks in a CT room as above.

**Table 1:** A* indicates a combination of originating host species and receiving host species that was studied in the experiment.

Host species from which psyllid originated	Host species on which psyllid was studied			
	Potato	Tomato	Poroporo	Boxthorn
Potato	*			
Tomato		*		
Poroporo	*	*	*	
Boxthorn	*	*		*

Adult psyllids from each non-crop host species were transferred to each crop host species. As controls, adult psyllids cultured on each host species were placed on the same species from which they originated. Eight treatments were evaluated using insects from the four host species (Table 1).

The ecological fitness was then evaluated by recording the growth rate and survival of immature life stages in each treatment. At 24h intervals, observations were made for the emergence of nymphs from the eggs (nymph eclosion), and the nymphs were observed for their rate of development to the emergence of adults (adult eclosion). Time from oviposition to adults’ emergence was also calculated (total development time). The number of nymphs that emerged from eggs was used to calculate the percentage of viable eggs and the number of adults that emerged provided a measure of nymphal survival. Total survival was calculated by analysing the percentage of eggs that survived through to the adult stage. These observations were made until the last adult emerged or all nymphs had died.

The data for development and survival were used to calculate means for each treatment. The data were analysed using two-way ANOVA (factors treatments and blocks) in GenStat 18th edition (VSN International) followed by an unrestricted 5%-level least significant difference (LSD) procedure, as recommended by Saville [29].

## Results

All TPP adults sampled for CLso were positive for the presence of the bacterium. The incubation period of eggs (nymph eclosion) from adults transferred from poroporo to tomato was 6.9 days (Table 2). This was statistically similar to poroporo to poroporo (8.2 days) but significantly lower (P < 0.05) than for tomato to tomato (9.0 days), on the basis of an unrestricted 5%-level LSD procedure. For boxthorn to tomato, the incubation period was 7.2 days; this was statistically similar to boxthorn to boxthorn (8.1 days) but significantly less (P < 0.05) than for tomato to tomato (9.0 days) (P < 0.05). For adults transferred from the three hosts poroporo, boxthorn and potato, to potato, the corresponding differences were not statistically significant. Nymph developmental time (adult eclosion) was similar for all treatments. Total development time (egg to adult) was substantially shorter for the progeny of adults from poroporo transferred to tomato (20.5 days) than those from tomato to tomato (23.2) (P < 0.05). There was no significant difference between the other treatments (Table 2).

**Table 2:** Development time (days) between life stages of tomato potato psyllid progeny from adults originating from the same or different host plant species. Within a column, means (n = 6) with a letter in common within a column did not differ significantly on the basis of an unrestricted 5%-level LSD procedure.

	Development time (days) between life stages					
Treatment	Nymph eclosion		Adult eclosion		egg to adult	
Boxthorn to boxthorn	8.1	ab	13.5	a	21.7	ab
Boxthorn to potato	8.0	ab	13.8	a	21.6	ab
Boxthorn to tomato	7.2	a	14.3	a	21.7	ab
Poroporo to poroporo	8.2	ab	13.5	a	21.8	ab
Poroporo to potato	7.6	ab	12.9	a	20.8	ab
Poroporo to tomato	6.9	a	13.7	a	20.5	a
Potato to potato	9.2	b	13.3	a	22.9	ab
Tomato to tomato	9.0	b	14.1	a	23.2	b
**LSD (5%)**	**1.8**		**2.5**		**2.5**	

Although not statistically significant, the mean percentage of eggs that developed into nymphs ranged from approximately 50% for progeny on potato when adults originated from boxthorn, to 87% for eggs on tomato when adults were from poroporo (Table 3). Adult emergence was lowest when both host plants were tomato (tomato to tomato, mean 50% emergence) and highest for poroporo to tomato (92%) (P < 0.05).

**Table 3:** Survival (percentage) between life stages of tomato potato psyllid progeny from adults originating from the same or different host plant species. Within a column, means (n = 6) with a letter in common within a column did not differ significantly on the basis of an unrestricted 5%-level LSD procedure.

	Survival (percentage) between life stages					
Treatment	Nymph eclosion		Adult eclosion		egg to adult	
Boxthorn to boxthorn	52.9	a	83.3	ab	40.7	a
Boxthorn to potato	49.8	a	80.4	ab	35.3	a
Boxthorn to tomato	81.4	a	73.1	ab	58.0	ab
Poroporo to poroporo	79.6	a	70.3	ab	51.1	ab
Poroporo to potato	59.7	a	60.3	ab	33.9	a
Poroporo to tomato	87.2	a	92.1	b	80.0	b
Potato to potato	71.5	a	70.2	ab	53.4	ab
Tomato to tomato	70.5	a	50.4	a	37.6	a
**LSD (5%)**	**38.5**		**33.1**		**38.2**	

For survival of eggs to adults, eggs on tomato from adults reared on poroporo again had the highest survival to adults eclosion (80%), which was significantly higher (P < 0.05) than those from boxthorn to boxthorn (41%), tomato to tomato (38%), boxthorn to potato (35%) and poroporo to potato (34%) (Table 3).

## Discussion

Some host plant species may not always be available to an insect throughout the year, especially for species that utilise annual plant species that are available for only part of the year. Therefore, the insect can survive on various well-distributed perennial non-crop host species, which are not always their preferred host species [30]. A previous study has shown that TPP prefers potato over other host species tested [31]. Additionally, potato was detected in the gut contents of TPP collected in autumn from a stand of wolfberry (*Lycium barbarum*), suggesting that some of the psyllids tested had developed, or at least fed on, potato prior to moving to the wolfberry [32]. It is widely believed that the source psyllid that infects annual potato crops are from perennial non-crop host species [32–34]. Such host plants may ultimately serve as refuges for the insect until the annual host species are available, to which some adults will migrate [6, 35]. Changing from one host species to another can have a negative or positive effect on the ecological fitness of the insect and its progeny [30]. The ability of progeny from migrating insects to survive on the new host species is essential for the insect to establish successfully [30].

In New Zealand, TPP populations develop on boxthorn and poroporo during the winter [16]. Although development at lower temperatures is slow [23], these plants are ideal for the insect to overwinter in small numbers. As temperature increases in spring and summer, adults disperse to annual crop host plants such as potato and tomato [16].

Previous studies have examined the development and survival of TPP on host plant species where the host plants of the adults were the same or different to that of the progeny [7, 8, 23, 26, 28, 36]. However, the impact on fecundity and survival of progeny of adults changing from one host plant species to another was not quantified. The present study considered reproductive females that developed on one host plant species ovipositing on another host species. The aim was to understand the impacts on the survival and development time of progeny of TPP adults that developed on a different host plant species.

In this study, eggs developed significantly faster on tomato when TPP females were transferred from boxthorn or poroporo compared to when the adult originated from tomato. The total development time of TPP from egg to adult for the different treatments followed a similar trend to nymph eclosion (Table 2).

Host plant quality impacts the oviposition of fertile eggs by an insect [26, 30]. Fertile eggs are those that produce nymphs [30]. When an insect selects a host plant for oviposition, it has determined that such a plant can provide food suitable for its offspring’s development. Additionally, when host plant suitability for development of offspring differs to the host plant that the adult developed on, eggs or ovarium may be resorbed and used as energy, or fewer eggs of higher quality may be produced [30, 37]. These high-quality eggs are likely to produce "fitter" offspring, increasing their chances of maintaining the species, therefore, improving ecological fitness [30]. Results obtained in the present study supported these conclusions to some extent because the development time of progeny was marginally less when their mothers changed host plant species (Table 2). This indicates that the offspring were of higher quality compared with the offspring of adults that did not change host plant species. However, the number of eggs produced by these migrating adults is needed to fully support this theory.

The reduced development period of TPP offspring when its mother was transferred from a non-crop host species to crop host species is new knowledge that can play a vital role in developing management strategies for this pest. However, the underlying reason for it is unclear, but could relate to a number of factors, including the impact of nutritional quality of the host plant on gut symbionts [38]. Our results indicate that the intrinsic rate of increase of the pest can be increased as opposed to if the insect population developed on the same host species over several generations. Although these results were obtained from a limited number of replicates of plants held in a controlled temperature room, these results are indicative that life table and forecasting models are needed for the pest on different host plants species. This study was only limited to two crop species and two non-crop host species. However, various factors including host species can influence the rate in which the pest can develop [26, 31]. Of the many listed non-crop host species of TPP [1, 15, 22], some are very localised [1]. Therefore, the use of information provided by developing life table and forecasting models for TPP progeny, must consider the natal host species distribution, given that the adults may move as much as 100 m per day [16]. Such models may help in developing and applying effective pest management strategies [39], such as when to apply agro-chemicals and help reduce the number of applications needed [40]. More research is needed for life table and forecasting models for TPP progeny on crop hosts after their mothers developed on a non-crop species.

Results obtained for the survival of nymphs did not show a similar pattern to those obtained for the development of nymphs to adults, where there was no statistical difference in development time between treatments. Instead, a notable difference was that nymphs on tomato from adults that developed on poroporo had a significantly higher survival rate than those of tomato to tomato. Additionally, more offspring from adults transferred from poroporo to tomato developed from eggs to adults, than those from tomato to tomato (Table 3).

Survival and development of an insect on a host species is also affected by the defence mechanism of said host species because plants contain various constitutive and induced defense mechanisms against insects [41]. If plant defence mechanisms were having an impact on the fitness of progeny, we would have expected progeny of adults from the same host plant species (i.e. tomato to tomato) to have had higher fitness than those from adults from different host plants. These results did not indicate that the host species defensive mechanism impacted the results, given that progeny of adults transferred from poroporo to tomato developed faster and survived better than for any other treatment. Those on tomato, of adults originating from tomato developed the slowest and survived the least among all treatments.

Field bindweed and black nightshade are listed as host plants of TPP [1, 15]. A previous study found bindweed to be an unsuitable host for the psyllid [34]. Since the progeny were unable to develop on these species in the present study, they may not be suitable as host species for the insect but rather, as casual plants. Casual plants are those on which the insect can feed and reproduce, but the progeny will not develop to adults [42]. Having these plants listed as host plants of TPP can cause some confusion and have economic and environmental implications especially in formulating a pest management protocol for the insect [22]. More research in different conditions is needed to ascertain if in fact, these are breeding host plants or not.

## Conclusion

The results from the present study indicate that, under laboratory conditions, TPP progeny of adults transferring from poroporo to tomato could have a faster development rate than those developing on the crop host species, which may thus accelerate population establishment in crop hosts after migrating to them. This indicates that, in these cases, the intrinsic rate of increase will be directly affected. Progeny of adults that transfer from boxthorn to a crop species are unlikely to incur any substantial fitness change relative to those that develop from adults on the same host species. However, further study is required, particularly through metabolomics and behavioural studies, to elucidate the underlying mechanisms that led to the results reported in the present study.

## Supporting information

S1 Data (XLSX)
